# Comprehensive diffusion tensor tractography of three dopaminergic pathways in advanced Parkinson’s disease: a case report

**DOI:** 10.1186/s12883-026-04757-0

**Published:** 2026-02-26

**Authors:** Heun Jae Ryu, Jeong Pyo Seo, Seo Yoon Park

**Affiliations:** 1 Department of Physical Therapy, College of Health Science, Honam University, Gwangju, 62399 Republic of Korea; 2 Department of Physical Therapy, College of Health and Welfare Sciences, Dankook University, 31116 Cheonan, Republic of Korea; 3 Department of Physical Therapy, College of Health and Welfare, Woosuk University, 443 Samnye-ro, Samnye-eup, 55338 Wanju-gun, Republic of Korea

**Keywords:** Parkinson's disease, Diffusion tensor tractography, Dopaminergic pathways, Mesocortical tract, Mesolimbic tract, Nigrostriatal tract, Case report

## Abstract

**Background:**

This qualitative, exploratory case report presents comprehensive diffusion tensor tractography (DTT) analysis of all three major dopaminergic pathways in advanced Parkinson’s disease (PD), which is rarely reported. We present a case demonstrating structural alterations across mesocortical, mesolimbic, and nigrostriatal pathways in a patient with severe motor and non-motor symptoms, highlighting DTT’s educational and clinical visualization value.

**Case presentation:**

A 77-year-old female with advanced PD presented with a complex symptom profile including severe cognitive impairment (MMSE not assessable), major depression (predating motor symptoms by 7 years), severe dysphagia with silent aspiration (PAS 8), bilateral motor weakness (MMT P- to P+), and wheelchair dependence. DTT analysis revealed marked architectural differences compared to an age-matched control subject. The mesocortical tract showed notably reduced fiber density and thinning, particularly in prefrontal cortex projections. The mesolimbic tract demonstrated diminished volume with altered trajectory patterns, especially affecting ventral tegmental area connections. The nigrostriatal tract exhibited the most pronounced degeneration with markedly reduced fiber density and widespread disruption of tract integrity.

**Conclusions:**

While qualitative in nature and limited by single-case design and comparison with a single age-matched control subject acquired on a different MRI system, this case illustrates comprehensive structural deterioration across three dopaminergic pathways in advanced PD, with pathway-specific structural features observed alongside the patient’s complex clinical presentation. This detailed visualization demonstrates DTT’s potential utility as a clinical tool for understanding both motor and non-motor manifestations in advanced PD. The findings highlight the educational value of comprehensive dopaminergic pathway assessment and suggest directions for future controlled studies.

## Introduction

Parkinson’s disease (PD) is a progressive neurodegenerative disorder characterized by the loss of dopaminergic neurons, particularly in the substantia nigra pars compacta [1]. This neurodegeneration affects three major dopaminergic pathways: the nigrostriatal tract (controlling motor function), the mesocortical pathway (involved in executive functions and cognition), and the mesolimbic pathway (crucial for reward and emotional processing) [2–4]. Understanding the structural integrity of these pathways is important for several reasons. While motor symptoms have traditionally been the focus of PD research, non-motor symptoms significantly impact patients’ quality of life and often precede motor manifestations [5]. Moreover, the complex interaction between these pathways influences both disease progression and treatment effectiveness [6].

Diffusion tensor tractography (DTT), derived from diffusion tensor imaging (DTI), enables three-dimensional visualization of specific neural pathways, providing insights into structural connectivity changes in neurological conditions [7, 8]. Several studies have demonstrated DTT’s utility in evaluating the nigrostriatal pathway in PD [9, 10], but comprehensive analyses simultaneously examining all three major dopaminergic pathways in advanced PD cases remain limited in the literature. The visualization of these pathways can provide valuable information about their architecture and connectivity patterns, particularly in advanced stages where severe structural changes might be present [11, 12].

Prior DTT studies in PD have predominantly focused on the nigrostriatal pathway, demonstrating reduced tract density and altered fractional anisotropy values in early to moderate disease stages [9, 10, 13]. Studies examining the mesocortical pathway have reported associations between tract integrity and cognitive decline in PD patients with dementia [14, 15], while investigations of the mesolimbic pathway have linked structural changes to affective symptoms and depression [16, 17]. However, comprehensive assessment of all three dopaminergic pathways simultaneously in a single advanced PD case remains notably absent from the literature, limiting our understanding of how these systems may be affected concurrently in end-stage disease.

Advanced PD cases presenting with severe multidomain symptomatology are rarely subjected to detailed DTT analysis, yet such cases offer important educational value and insights into end-stage structural changes. This visualization approach could enhance understanding of the relationship between structural alterations and clinical manifestations, potentially aiding clinicians in comprehending complex symptom profiles [11].

We report a case of advanced PD where DTT was used to visualize and characterize alterations in all three major dopaminergic pathways. This comprehensive approach illustrates the structural basis of the patient’s complex symptomatology, demonstrating DTT’s potential utility as a clinical visualization and educational tool. Importantly, this case report does not aim to infer or establish structure–function relationships, but rather to provide an anatomical visualization framework illustrating pathway-specific architectural changes in advanced PD.

## Case report

### Clinical history

A 77-year-old female patient presented with advanced Parkinson’s disease that showed a progressively deteriorating course. The patient was initially diagnosed with Parkinson’s disease in April 2016, when she began using a walking aid intermittently. Her medical history was significant for major depression and alcohol use disorder since 2009, and she had been diagnosed with dementia in 2013 prior to her Parkinson’s disease diagnosis. Sixteen months after onset, she suffered a left femoral neck fracture requiring total hip replacement, after which she became wheelchair-dependent. By November 2019, her functional status had deteriorated significantly, reaching a modified Rankin Scale score of 4.

### Motor profile

At the time of evaluation in May 2023, bilateral motor weakness was evident, with Manual Muscle Testing showing P- to P+ level in both upper and lower extremities. Physical examination revealed preserved pupillary reflexes. Despite severe impairment, the patient maintained some functional abilities, including the capacity to follow single-step commands and respond to name questions with lip movements. She demonstrated reflexive swallowing within 30 s after oral stimulation and could maintain a standing position for one minute with full assistance.

### Cognitive and psychiatric symptoms

The patient presented with severe hypersomnia, although recent adjustments had improved her daytime wakefulness. She also exhibited hyperventilation, which showed improvement with dopaminergic medication adjustment. Her cognitive function was severely impaired, rendering the Mini-Mental State Examination impossible to conduct.

### Swallowing function

Videofluoroscopic swallowing examination revealed severe dysphagia with silent aspiration (PAS 8) for both thin liquids and semisolids.

### Comorbidities

The patient’s condition was further complicated by multiple comorbidities, including hypertension, type 2 diabetes mellitus, unspecified dementia, atrial fibrillation, suspected deep vein thrombosis, and bilateral osteoarthritis (Kellgren-Lawrence grade 4/4).

### Functional status and treatment response

During the course of treatment, the patient showed modest improvements in alertness and ventilation with medication adjustment and rehabilitation efforts. Her responses to dopaminergic medication were particularly notable in managing hyperventilation symptoms, suggesting preserved responsiveness in certain dopaminergic pathways despite advanced disease. The rehabilitation program focused on maintaining functional abilities through physical and occupational therapy, resulting in limited but meaningful achievements such as assisted standing capability.

Given her complex medical conditions and advanced disease state, a comprehensive follow-up plan was established, including continued care in rehabilitation medicine, internal medicine, otolaryngology, and neurology departments. The patient was scheduled for transfer to a long-term care facility for ongoing management of her condition. Regular monitoring of cognitive status, swallowing function, and motor abilities was planned to optimize her care regimen and quality of life. Written informed consent was obtained from the patient’s legal guardian for the publication of this case report and accompanying neuroimaging results. The patient’s privacy and personal information have been protected in accordance with ethical guidelines.

## Methods

### DTI data acquisition

DTI data acquisition for the patient was performed in May 2023. DTI data were acquired using a Synergy-L SENSE head coil on a 3T system equipped with single-shot echo-planar imaging. For each of the 32 non-collinear diffusion-sensitizing gradients, 67 contiguous slices were acquired parallel to the anterior commissure-posterior commissure line. The imaging parameters were as follows: acquisition matrix = 96 × 96, reconstructed matrix = 192 × 192 matrix, field of view = 240 × 240 mm², TR = 10,398 ms, TE = 72 ms, parallel imaging reduction factor (SENSE factor) = 2, EPI factor = 59 and b = 1000 s/mm², NEX = 1, slice gap = 0, and slice thickness 2.5 mm. The age-matched control subject was scanned using a 1.5T Gyroscan Intera system (Philips, Best, The Netherlands) with comparable imaging parameters.

### Image processing and tractography

The Oxford Centre for Functional Magnetic Resonance Imaging of the Brain (FMRIB) software library (FSL; www.fmrib.ox.ac.uk/fsl) was used to analyze the diffusion-weighted imaging data [18, 19]. Affine multi-scale two-dimensional registration was used to correct for head motion effects and image distortions caused by eddy currents. A probabilistic tractography method based on a multi-fiber model was used for fiber tracking and was applied utilizing tractography routines implemented in FMRIB’s Diffusion Toolbox (step length 0.5 mm, 5000 streamline samples, curvature threshold = 0.2) [19–22].

### Dopaminergic pathway reconstruction

Three major dopaminergic pathways were reconstructed following established protocols [23–25]:

1. *Mesocortical tract*: Fibers connecting the ventral tegmental area to the prefrontal cortex. Two regions of interest (ROIs) were placed: the first ROI at the ventral tegmental area in the midbrain, and the second ROI in the prefrontal cortex [23].
2. *Mesolimbic tract*: Fibers from the ventral tegmental area to the nucleus accumbens. ROIs were placed at the ventral tegmental area and the nucleus accumbens region in the ventral striatum [23].
3. *Nigrostriatal tract*: Fibers connecting the substantia nigra to the striatum. ROIs were placed at the substantia nigra in the midbrain and the dorsal striatum [24, 25].

ROI placement was independently verified by two experienced researchers with expertise in neuroimaging to ensure anatomical accuracy and consistency. ROI placement was performed by two experienced researchers who were blinded to the diagnostic status of the subjects to minimize potential bias. The same probabilistic tractography parameters and threshold values were applied consistently for both the patient and control subject. For optimal visualization of all dopaminergic pathways, fiber tracking was performed with a threshold value of 3, which was selected to maximize tract visibility while maintaining anatomical plausibility.

### Qualitative comparison analysis

The reconstructed dopaminergic pathways were qualitatively compared with those of an age-matched healthy control subject (77-year-old female with no history of neurological or psychiatric disorders). The control subject is used solely for illustrative visual comparison and does not constitute a normative reference or semi-quantitative comparator. Inter-individual variability in tractography reconstruction, particularly in elderly brains, limits direct comparisons between individuals. The control subject had no known history of microvascular disease, though age-related microvascular changes cannot be entirely excluded. This comparison focused on visual assessment of the following features:

- Tract presence and overall continuity.
- Architectural patterns and trajectory.
- Relative fiber density (assessed through visual inspection of tract volume and coverage).
- Pathway integrity and coherence.

No quantitative statistical analysis was performed given the single-case design. The comparison was intended to illustrate gross architectural differences and provide context for interpreting the patient’s structural changes, rather than to establish statistical significance. Observations were documented through consensus between the two independent reviewers to enhance reliability of the qualitative assessments. We acknowledge that the patient was scanned at 3T while the control was scanned at 1.5T. Importantly, since 3T imaging typically provides superior tract visualization compared to 1.5T, the observation of markedly reduced tract architecture in the patient (scanned at 3T) compared to the control (scanned at 1.5T) represents a conservative finding, as any scanner-related bias would favor better visualization in the patient.

## Results

DTT analysis was performed in a 77-year-old female patient with advanced Parkinson’s disease and compared with an age-matched healthy control subject (77-year-old female with no neurological history). Visual comparison revealed notable architectural differences across all three major dopaminergic pathways.

### Mesocortical tract

The mesocortical tract showed marked structural alterations compared to the control subject. The patient’s tract exhibited notably reduced fiber density and thinning, particularly evident in projections to the prefrontal cortex. Lateral view analysis revealed disruption in tract continuity, with fragmentation of the pathway architecture that was distinctly different from the continuous, well-organized tract observed in the control subject. These structural differences were observed alongside the patient’s severe cognitive impairment, including inability to complete MMSE assessment and difficulty with multi-step commands, though a causal relationship cannot be confirmed from this single-case qualitative analysis.

### Mesolimbic tract

The mesolimbic tract demonstrated considerable degradation in the patient compared to the control subject. The overall tract volume appeared diminished, with alterations in pathway integrity and trajectory patterns. The pathway architecture showed reduced coherence, particularly in connections involving the ventral tegmental area, contrasting with the robust, well-defined tract observed in the control. This structural deterioration was observed in parallel with the patient’s history of major depression, which predated her motor symptom onset by seven years, although multiple factors may contribute to this association.

### Nigrostriatal tract

The nigrostriatal tract revealed the most pronounced degeneration among the three pathways. The patient showed markedly reduced fiber density throughout the tract, with widespread disruption of pathway integrity. The extent of architectural changes was substantial when compared to the control subject’s well-preserved nigrostriatal tract, which displayed continuous fibers with clear definition. This pattern of severe structural alteration was observed alongside the patient’s severe motor symptoms, including bilateral motor weakness (MMT P- to P+) and progression to wheelchair dependence.

### Comparative overview

**Fig. 1:**
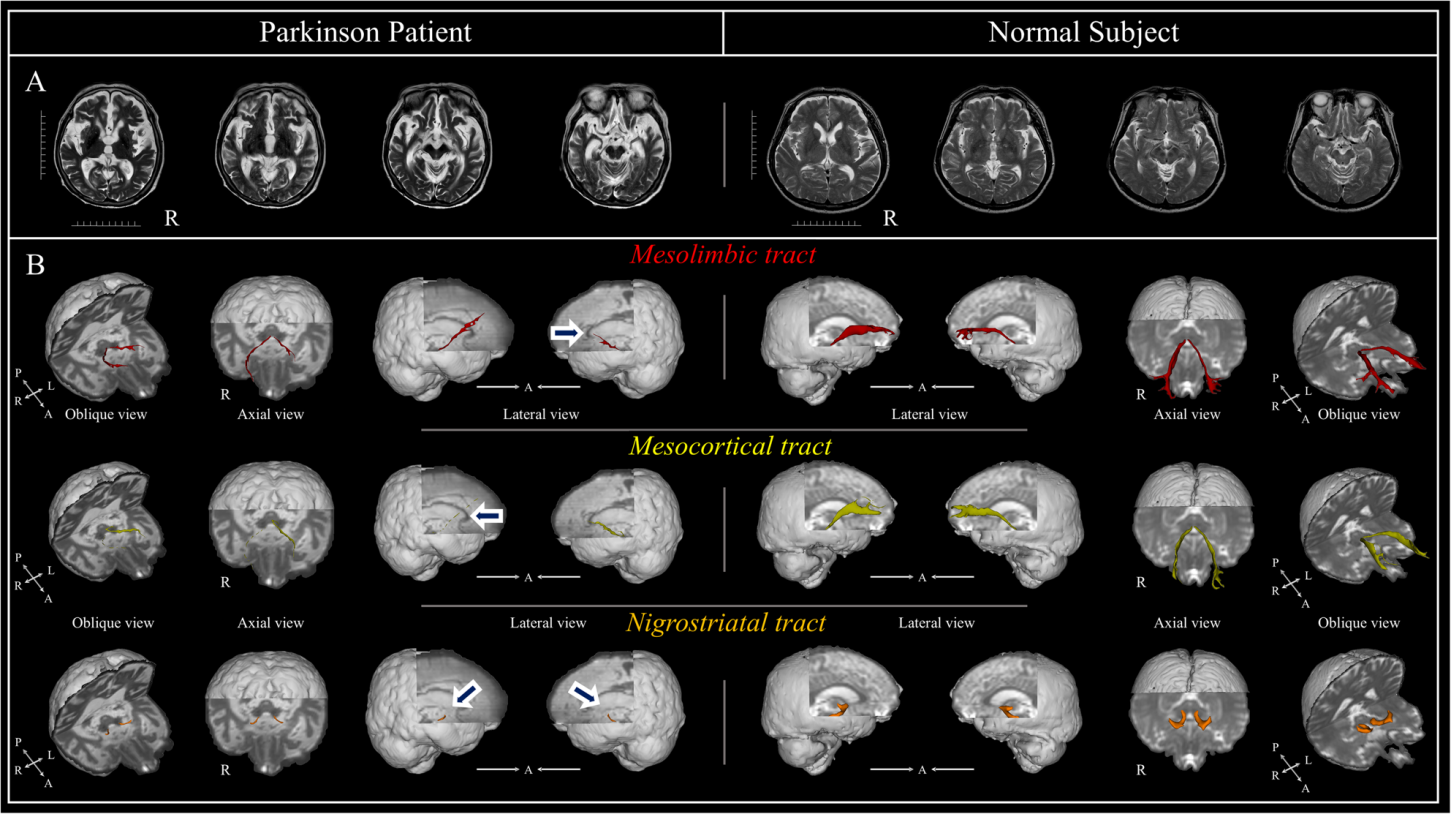
DTT visualization of major dopaminergic pathways in Parkinson's disease and normal control. **A**. T2-weighted axial brain MRI images showing anatomical structures at the same brain levels in Parkinson's patient (left) and normal subject (right). **B**. DTT reconstructions showing three major dopaminergic pathways: • Mesolimbic tract (red): Patient shows reduced volume and altered trajectory (blue arrows) compared to normal subject's intact pathway. • Mesocortical tract (yellow): Patient exhibits reduced fiber density and thinning (blue arrows), especially in prefrontal projections. • Nigrostriatal tract (orange): Patient demonstrates severe degeneration with reduced fiber density (blue arrows) versus normal subject's intact architecture. Images shown in oblique, axial, and lateral views for each tract. R: right, A: anterior, L: left, P: posterior. Scale bar = 100 mm (10 mm intervals)

Collectively, all three dopaminergic pathways demonstrated notable structural compromise in the patient, with the severity of apparent degeneration most evident in the nigrostriatal tract, followed by the mesocortical and mesolimbic pathways. The pathway-specific patterns of structural change were observed alongside the patient’s clinical symptom profile, encompassing motor, cognitive, and neuropsychiatric manifestations (Fig. 1). 

## Discussion

### Main findings and clinical significance

This case report presents comprehensive diffusion tensor tractography analysis of all three major dopaminergic pathways in advanced Parkinson’s disease. The simultaneous visualization revealed distinct patterns of structural alteration observed alongside the patient’s complex clinical presentation, illustrating DTT’s potential as a visualization and educational tool for understanding both motor and non-motor manifestations in advanced PD.

The significance of this case lies in three aspects: (1) detailed neuroimaging documentation of end-stage structural changes in a patient with severe multidomain symptoms, (2) holistic assessment of all three dopaminergic pathways rather than focusing solely on the nigrostriatal system, and (3) descriptive documentation of pathway-specific architectural patterns alongside corresponding clinical features.

### Pathway-specific observations and clinical correlations

#### Mesocortical tract

The marked reduction in fiber density and thinning, particularly in prefrontal projections, was observed alongside the patient’s severe cognitive impairment (unmeasurable MMSE, difficulty following commands). This aligns with previous studies demonstrating associations between mesocortical pathway integrity and cognitive function in PD [14, 15]. Our case illustrates the extent of pathway deterioration possible in advanced stages, providing structural features observed in parallel with severe cognitive decline.

#### Mesolimbic tract

The notably diminished volume and altered trajectory were observed in parallel with the patient’s depression history, which preceded motor symptoms by seven years. This temporal pattern supports emerging literature emphasizing non-motor symptoms as early disease markers [16, 17], potentially offering structural perspective on the well-documented relationship between depression and PD.

#### Nigrostriatal tract

The severe degeneration with markedly reduced fiber density was observed alongside severe motor symptoms—bilateral weakness (MMT P- to P+) and progression to wheelchair dependence. The extent of deterioration appears consistent with advanced disease stage and aligns with quantitative DTI studies demonstrating progressive microstructural degeneration [10, 13].

#### Comparison with literature

While previous DTT studies primarily focused on the nigrostriatal pathway or examined pathways in isolation [9, 10], our simultaneous analysis of all three systems provides more comprehensive structural perspective. The observed pattern—most severe changes in nigrostriatal tract, followed by mesocortical and mesolimbic alterations—aligns with known PD pathology progression [1, 3]. The severity of changes in our advanced-stage patient exceeds what is typically reported in early or moderate PD studies, highlighting the progressive nature of white matter degeneration. Quantitative DTI studies have reported decreased fractional anisotropy values in the substantia nigra of PD patients compared to controls, with progressive decline correlating with disease duration [10, 13]. Studies examining cognitive decline in PD have demonstrated mesocortical pathway involvement, with white matter changes in the cingulum bundle associated with dementia development [14, 15]. The mesolimbic pathway has been implicated in PD-associated depression, with structural alterations in reward circuitry correlating with affective symptom severity [16, 17].

The pattern of pathway involvement observed in our patient—with widespread degeneration across all three dopaminergic systems— may be interpreted within the conceptual framework of Braak’s staging model of PD pathology [1]. In Braak stages 5–6, pathological changes are described as extending beyond the substantia nigra to affect limbic and neocortical structures, which would be expected to manifest as concurrent involvement of nigrostriatal, mesolimbic, and mesocortical pathways. However, this interpretation remains speculative in the absence of longitudinal imaging or neuropathological confirmation, and the Braak model is presented here solely as a conceptual reference rather than as evidence supporting our observations.

#### Limitations and interpretive cautions

##### Single-case design

Without larger cohort, we cannot establish statistical significance or generalizability. Findings should be viewed as hypothesis-generating. Future controlled studies with adequate sample sizes are essential to validate these observations. The use of a single cross-sectional scan represents an important limitation, as it prevents assessment of temporal changes in tract integrity and precludes determination of whether structural alterations preceded or followed specific symptom development. Longitudinal imaging studies would be necessary to establish the temporal relationship between tract degeneration and clinical symptom progression.

##### Single age-matched control

Limits our ability to distinguish normal aging variation from pathological changes. The use of a single control subject scanned on a different MRI system (1.5T vs. 3T) constitutes a structural limitation that restricts interpretability of the qualitative comparison, even considering the conservative nature of the observed differences. However, our observations align with patterns reported in quantitative studies using appropriate control groups [13, 14], suggesting major architectural differences likely represent genuine pathological changes.

##### Multiple comorbidities

Pre-existing dementia (2013) and depression (2009) complicate interpretation. We acknowledge that the observed tract alterations cannot be solely attributed to Parkinson’s disease. Importantly, given the patient’s pre-existing dementia (diagnosed three years before PD) and longstanding depression and alcohol use disorder (predating PD by seven years), the observed alterations in mesocortical and mesolimbic pathways cannot be primarily attributed to Parkinson’s disease. These comorbid conditions are independently associated with white matter changes in the same pathways [26, 27], and their relative contributions cannot be disentangled in this single case. Interpretation of non-nigrostriatal pathway changes should therefore remain particularly cautious. Only the nigrostriatal pathway changes, which showed the most pronounced degeneration, can be more confidently associated with PD pathology, given that the temporal sequence follows typical PD progression and responsiveness to dopaminergic medication indicates functional pathway involvement.

##### Qualitative analysis

Visual assessment lacks precision of quantitative metrics. Assessments performed through consensus between experienced researchers enhance reliability, but subjective interpretation remains a limitation. Future studies should incorporate standardized quantitative metrics.

### Technical limitations

DTT reconstruction of dopaminergic pathways presents several inherent technical challenges that warrant consideration [26, 28]. Probabilistic tractography, while advantageous for handling uncertainty in fiber orientation, is sensitive to noise and tracking parameters, and may produce variable results depending on threshold selection and seed placement. The fixed tractography threshold (threshold = 3) used for visualization, while selected to maximize tract visibility while maintaining anatomical plausibility, represents an arbitrary parameter choice that constitutes an additional source of potential variability. The midbrain region where dopaminergic pathways originate contains complex crossing fiber configurations that can affect reconstruction accuracy, particularly for the mesolimbic and mesocortical tracts which share common origin in the ventral tegmental area.

We acknowledge that our patient was imaged at 3T while the control subject was scanned at 1.5T. Higher field strength (3T) typically provides improved signal-to-noise ratio and better angular resolution for diffusion imaging, which generally results in superior tract visualization. Therefore, the observation of markedly reduced tract architecture in our patient—despite the technical advantage of 3T imaging—represents a conservative finding. Any scanner-related bias would favor better visualization in the patient, suggesting that the observed differences likely reflect genuine pathological changes rather than technical artifacts. Expected inter-individual variability in tract shape and density, which has been documented even among healthy controls [27], should also be considered when interpreting qualitative comparisons. Despite these limitations, the gross anatomical patterns observed are unlikely to be solely attributable to technical factors, particularly given their parallel observation alongside the patient’s clinical symptoms.

### Clinical and educational implications

This comprehensive visualization approach has potential applications in aiding clinicians’ understanding of the structural basis of complex symptom profiles and in providing educational value for multidisciplinary teams and patient/family communication. While our qualitative analysis cannot provide quantitative metrics for clinical decision-making, it demonstrates proof-of-concept for pathway-specific assessment. Future development of standardized DTT protocols and normative databases could translate these observations into clinically useful tools.

## Conclusion

Rather than establishing structure–function relationships, this case provides an anatomical visualization framework that illustrates the extent of pathway-specific structural deterioration in advanced PD. This case illustrates comprehensive structural deterioration across three dopaminergic pathways in advanced PD, with pathway-specific structural features observed alongside complex clinical presentation. While qualitative in nature and limited by single-case design, and comparison with a single age-matched control subject acquired on a different MRI system, this detailed visualization contributes to understanding the structural basis of multidomain symptomatology in advanced PD. The parallel observations of structural alterations and clinical symptoms suggests DTT’s value as an educational tool and potential future clinical application. Larger studies with quantitative methods are needed to validate these observations and establish standardized protocols for clinical implementation.

## Data Availability

The datasets used and analyzed during the current study are available from the corresponding author on reasonable request.

## Abbreviations

DTT: Diffusion Tensor Tractography
DTI: Diffusion Tensor Imaging
PD: Parkinson’s Disease
MMSE: Mini-Mental State Examination
MMT: Manual Muscle Testing
PAS: Penetration-Aspiration Scale
ROI: Region of Interest
TR: Repetition Time
TE: Echo Time
NEX: Number of Excitations
FMRIB: Functional Magnetic Resonance Imaging of the Brain
FSL: FMRIB Software Library
